# Can eosinophilia and neutrophil–lymphocyte ratio predict hospitalization in asthma exacerbation?

**DOI:** 10.1186/s13223-021-00512-x

**Published:** 2021-02-10

**Authors:** Hossein Esmaeilzadeh, Fatemeh Nouri, Seyed Hesamodin Nabavizadeh, Soheila Alyasin, Negar Mortazavi

**Affiliations:** 1 Allergy Research Center, Shiraz University of Medical Sciences, Shiraz, Iran; 2 Department of Allergy and Clinical Immunology, Namazi Hospital, Shiraz University of Medical Sciences, Shiraz, Iran; 3 Student Research Committee, Shiraz University of Medical Sciences, Shiraz, Iran; 4 Department of Clinical Pharmacy, Shiraz University of Medical Sciences, Shiraz, Iran

**Keywords:** Asthma, Asthma exacerbation, Eosinophil, Asthma exacerbation, Neutrophil–lymphocyte ratio, Blood eosinophil count

## Abstract

**Objective:**

Asthma is one of the most common diseases amongst children. Blood eosinophil count and neutrophil–lymphocyte ratio (NLR) are known as markers for phenotyping asthma. This study was performed to investigate blood eosinophil count and NLR as predictors of hospitalization in pediatric asthma exacerbations.

**Data sources and study selections:**

In this cross-sectional study, children admitted to hospital ward for more severe asthma exacerbation were compared with non-hospitalized children with moderate to severe asthma exacerbation whose asthma exacerbation was managed in emergency department or outpatient clinic. We investigated patients’ characteristic and factors associated with hospitalization.

**Results:**

A total of 211 children with moderate to severe asthma exacerbation (mean age $5.76 \pm 3.28$ years old) were enrolled in the study including 91 hospitalized patients and 120 non-hospitalized patients. For the prediction of hospitalization, an ROC Curve analysis was performed and revealed a cut-off of 298 cells/µL and 2.52 of blood eosinophil count and NLR, respectively. In multivariate analysis, not using an asthma action plan (OR 2.22, 95% CI 1.09–4.49; *P* = 0.027), a blood eosinophil count $\ge$ 298 (OR 8.79, 95% CI 4.44–17.4; *P* < 0.001) and an NLR $\ge$ 2.52 (OR 2.13, 95% CI 1.09–4.14; *P* = 0.027) were associated with hospitalization.

**Conclusion:**

Blood eosinophil count and NLR were found to be higher in hospitalized children with more severe asthma exacerbation compared to non-hospitalized patients. These markers can be indicators for asthma exacerbation severity.

## Key message

Since in hospitalized children with asthma exacerbation, blood eosinophil count and NLR were observed to be higher than non-hospitalized ones, these markers can help predicting hospitalization in paediatric patients with asthma exacerbation.

## Background

Asthma is one of the most common chronic disease in children [1]. According to a CDC report in 2006, asthma lead to approximately 200,000 hospitalizations per year [2]. Rates of hospital admissions and ED visits are higher among moderate to severe asthma patients with exacerbations compared to those without exacerbations [3].

Asthma is a chronic inflammatory disease of the airways that has different clinical and immunological phenotypes. To date, two cluster analyses were done in Severe Asthma Research Program (SARP) for characterization of different presentations of severe asthma. The first cluster analysis included clinical and physiologic parameters (e.g., lung function, atopy), while bronchoscopic and inflammatory data (e.g. bronchoalveolar lavage and blood cell counts, exhaled nitric oxide) were main variables of the second one. Different phenotypes like early onset asthma, allergic asthma (mild, moderate, and severe) with an inflammatory process in severe types were recognized in both analyses [4, 5]. Evidence suggest that eosinophils and neutrophils are major cells contributing to asthma pathology, and neutrophils are more prevalent in severe asthma [4–6]. Asthma severity is known to be correlated with the sputum neutrophil counts, level of blood eosinophils, and the bronchoalveolar fluid cells [7, 8].

Asthma exacerbation is a worsening in condition of a patient with stable asthma, which is mostly caused by viral infection [9], and hospitalizations due to severe exacerbations mostly happen in moderate to severe persistent asthma [10]. Blood eosinophils and neutrophils are both associated in occurrence of asthma exacerbations. The latest studies have suggested that number of eosinophils in blood and sputum can predict asthma exacerbation [11, 12]. A high level of eosinophils in the blood can also be taken as a risk factor for exacerbations of asthma [13–16]. Understanding inflammatory processes of exacerbations can lead to development of strategies for prevention and treatment.

Neutrophil–lymphocyte ratio (NLR) can indicate systemic inflammation. Mean NLR increases in asthma patients especially in those with unstable asthma compared to healthy subjects [17, 18]. Regarding the effect of inflammation on asthma and its worsening in exacerbations, NLR assay can be helpful in differentiation of asthma exacerbations.

Based on guidelines of the Expert Panel Report 3 (EPR-3), severity of an asthma exacerbation is determined by signs and symptoms such as respiratory distress, dyspnea, Initial Peak Expiratory flow (PEF), oxygen saturation, and clinical course. Furthermore, patients with severe respiratory distress, low oxygen saturation and not responsive to primary care with oxygen, beta-agonists, and systemic glucocorticoids should be admitted in hospital ward [19].

To the best of our knowledge, there is no study simultaneously evaluating blood eosinophil and NLR in asthma exacerbation. Thus, the present study was conducted to investigate these markers as biomarkers of asthma exacerbation severity and specifically as contributing factors for hospital admission.

## Methods

### Study design, population, and data source

We carried out a retrospective cross-sectional study on children aged less than 18 years old with moderate to severe asthma exacerbation classified based on Expert Panel Report 3 [19] and referred to Namazi tertiary referral hospital and Imam-Reza outpatient clinic in Shiraz, southwest of Iran during 2014–2019. Ethical approval for our study was obtained from ethics committee of Shiraz University of Medical Sciences. Data were extracted from medical records and remained confidential. Before treatment of the exacerbation, an informed consent was obtained from participants’ parents. Subjects were included in 2 groups: first group involved the patients who were admitted to the ward (hospitalized) due to life threatening or moderate to severe asthma exacerbation not responding to rescue therapy (oxygen, short acting β-agonist, and systemic corticosteroid in the first hours) [19], and second group consisted of patients with moderate to severe asthma exacerbations who did not need to be hospitalized.

Data on patients’ demographic characteristics e.g. age, gender, complete blood count, white blood cell differential; and hospital course e.g. duration of admission, comorbidity, antibiotic consumption were collected (Additional file 1).

The patients were excluded if the asthma exacerbation was mild or CBC and eosinophil were influenced by medications and concomitant disorders like long-term systemic corticosteroid use or parasite infection.

### Statistical analyses

Categorical variables were addressed by *n* (%), while mean and standard deviation were used to present continuous values. Cross-sectional administrative data were analysed in a case control manner. Data were compared in hospitalized patients with non-hospitalized patients, and comparisons were also made in hospitalized patients in terms of blood eosinophil counts and NLR levels. Statistical analyses were done using receiver operating characteristic (ROC) Curve, ANOVA, Chi-Squared, Fisher’s exact test, Wilcoxon-signed rank, Mann–Whitney U, or Independent Samples T-tests as appropriate. Spearman’s correlation coefficient was used for assessing correlations between the variables. A binary logistic regression model was applied to determine the predictors of hospitalization in asthma exacerbation. The variables with a $P$ $< 0.2$ in univariate analysis were subsequently analysed using multivariate analysis to determine independent factors predicting hospitalization. Odds ratios (OR) and 95% confidence intervals (CI) are reported as appropriate. All analyses were done using SPSS software, version 16.0 (SPSS Inc., Chicago, Illinois). All comparisons were two-tailed, and a $P$ $< 0.05$ was considered as statistically significant.

## Results

### Patients’ characteristics

In this study, 28 out of 239 cases were excluded due to concomitant disorders and long-term corticosteroid use. 211 patients with moderate to severe asthma exacerbation (91 hospitalized and 120 not) with mean age of $5.76 \pm 3.28$ years old were included based on inclusion criteria, 124 (58.7%) of them were boys and 87 (41.2%) were girls. Clinical and demographic features of patients are shown in Table 1. Hospitalized patients were significantly younger than non-hospitalized ones (*P* = 0.020). Newly diagnosed asthma was significantly higher (28.6% vs. 15.8%, *P* = 0.025) and using a written action plan before exacerbation was significantly lower (55.6% vs. 72.9%, *P* = 0.005) among hospitalized patients.

**Table 1:** Comparison of characteristics in hospitalized and non-hospitalized patients

	Non-hospitalized	Hospitalized	*P*
	*n* = 120	*n* = 91	
Gender			0.788
Female, *n* (%)	51 (42.5)	36 (39.6)	
Male, *n* (%)	69 (57.5)	55 (60.4)	
Age (mean years ± SD)	$6.21 \pm 3.14$	$5.16 \pm 3.36$	0.020
Antibiotic consumption, *n* (%)	23 (19)	55 (60.4)	< 0.001
Using an asthma action plan^a^, *n* (%)	86 (72.9)	50 (55.6)	0.009
Presence of atopic condition^b^, *n* (%)	38 (32.7)	32 (35.6)	0.674
Cause of the exacerbation			
Infection, *n* (%)	73 (60.8)	64 (70.3)	0.152
Allergen, *n* (%)	11 (9.2)	5 (5.5)	0.323
Non-adherence to prescribed therapy, *n* (%)	28 (23.3)	19 (20.9)	0.671
Other, *n* (%)	8 (6.7)	3 (3.3)	0.275
Duration of asthma			
Newly diagnosed, *n* (%)	19 (15.8)	26 (28.6)	0.025
Less than 1 year, *n* (%)	38 (31.7)	22 (24.2)	0.232
1–2 years, *n* (%)	36 (30)	28 (30.8)	0.904
More than 2 years, *n* (%)	27 (22.5)	15 (16.4)	0.278
Blood eosinophil count (mean ± SD)	$260.4 \pm 301.7$	$573.3 \pm 368$	< 0.001
NLR (mean ± SD)	$2.29 \pm 2.5$	$4.96 \pm 4.27$	< 0.001

^a^Data missing for three patients

^b^History of allergic rhinitis and atopic dermatitis was considered as positive history of atopy. Data missing for five patients

During hospitalization course, antibiotics were prescribed for 55 cases (60.4%) of hospitalized patients. Among which, 47 cases (85.4%) received at least one dose of parentral antibiotic, 20 cases (36.7%) received oral macrolides and 17 patients (30.9%) received both parentral and oral antibiotics. Antibiotic therapy was significantly higher in hospitalized patients (*P* < 0.001). Mean hospital admission days were $3.81 \pm 3.15$ days.

### Blood eosinophil count

**Fig. 1:**
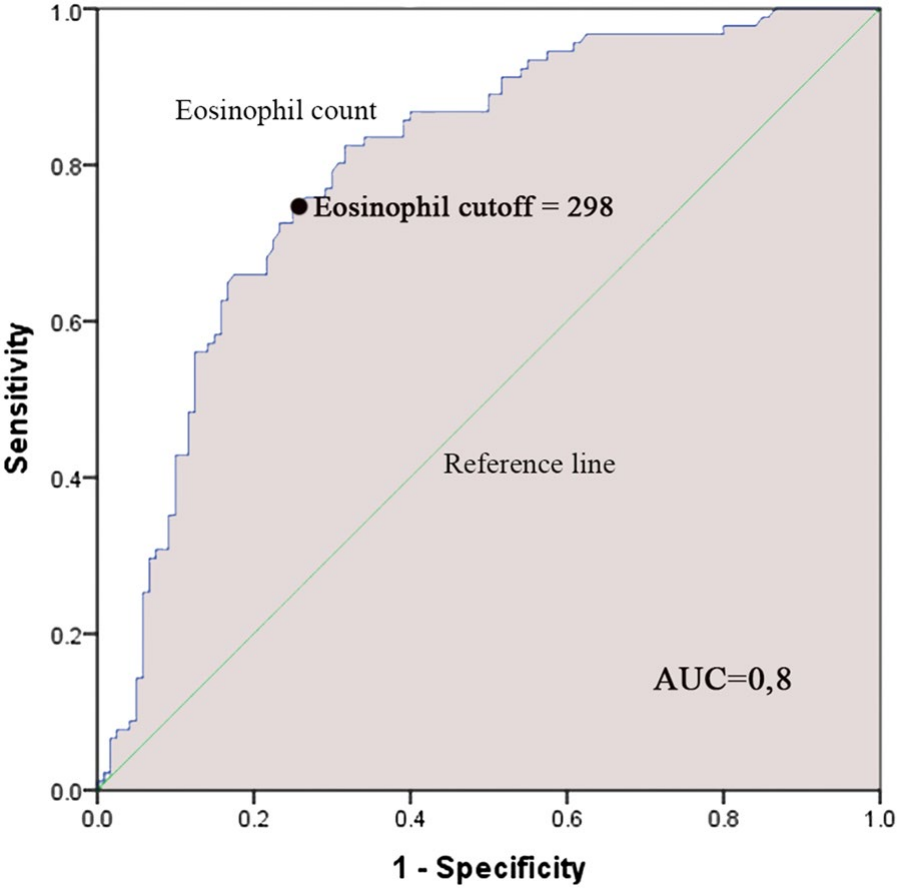
ROC Curve of blood eosinophil count predictor cutoff of hospitalization of moderate to severe asthma pediatric patients. The ROC curve shows that the predictor cutoff of blood eosinophils for hospital admission is 298 cells/µL. Area under the ROC curve was 0.8

Mean blood eosinophil count in hospitalized and non-hospitalized patients was significantly different ($573.3 \pm 368$ cells/µL vs $260.4 \pm 301.7$ cells/µL respectively, P < 0.001). The predictor cutoff of blood eosinophils for hospitalization was 298 cells/µL (AUC 0.8, sensitivity 74.7%, specificity 75%) using the ROC curve analysis (Fig. 1) which divided the patients to a group of 98 with a higher blood eosinophil count than cutoff and another consisted of 113 patients with a blood eosinophil count < 298 cells/µL.

There was no significant difference in blood eosinophil count between hospitalized and non-hospitalized patients receiving antibiotics ($579.1 \pm 384.6$ cells/µL vs. $565.3 \pm 348.6$ cells/µL, $P = 0.894$). Moreover, in the hospitalized group, 7 patients (7.7%) had concomitant pneumonia. Mean blood eosinophil count was lower in hospitalized patients with concomitant pneumonia than patients who did not ($427.6 \pm 227.9$ cells/µL vs. $585.5 \pm 375.7$ cells/µL, $P = 0.326$).

Demographic characteristics and laboratory data for admitted patients with blood eosinophil count of $\ge 298$ cells/µL and those with blood eosinophil count of less than 298 cells/µL are provided in Table 2.

**Table 2:** Characteristics of hospitalized patients based on blood eosinophil count and neutrophil–lymphocyte ratio

	Eosinophil count		*P*	NLR		*P*
	$\ge 298$	$< 298$		$\ge 2.52$	< 2.52	
	*n* = 68	*n* = 23		*n* = 53	*n* = 38	
Patients’ demographic characteristics						
Gender			0.149			0.911
Female, *n* (%)	26 (28.6)	5 (5.5)		20 (22)	11 (12.1)	
Male, *n* (%)	42 (46.2)	18 (19.8)		38 (41.8)	22 (24.2)	
Age (mean years ± SD)	5.25 $\pm 3.74$	$4.9 \pm 3.38$	0.833	$6.02 \pm 3.36$	$3.67 \pm 3.68$	< 0.001
Hospital course						
Antibiotic therapy, *n* (%)	48 (52.7)	15 (16.5)	0.63	39 (42.9)	24 (26.4)	0.586
Admission days (mean ± SD)	$3.82 \pm 3.37$	$3.78 \pm 2.45$	0.426	$3.72 \pm 3.45$	$3.97 \pm 2.58$	0.199
Eosinophil count (mean ± SD)	$688.9 \pm 344.1$	$140.8 \pm 86.6$		$540.7 \pm 377.4$	$259.4 \pm 297.1$	< 0.001
NLR (mean ± SD)	$5.2 \pm 3.9$	$1.9 \pm 2.5$	< 0.001	$6.1 \pm 3.62$	$0.97 \pm 0.65$	
Associated pneumonia, *n* (%)	3 (3.3)	4 (4.4)	0.672	2 (2.2)	5 (5.5)	0.124

### Neutrophil–lymphocyte ratio

**Fig. 2:**
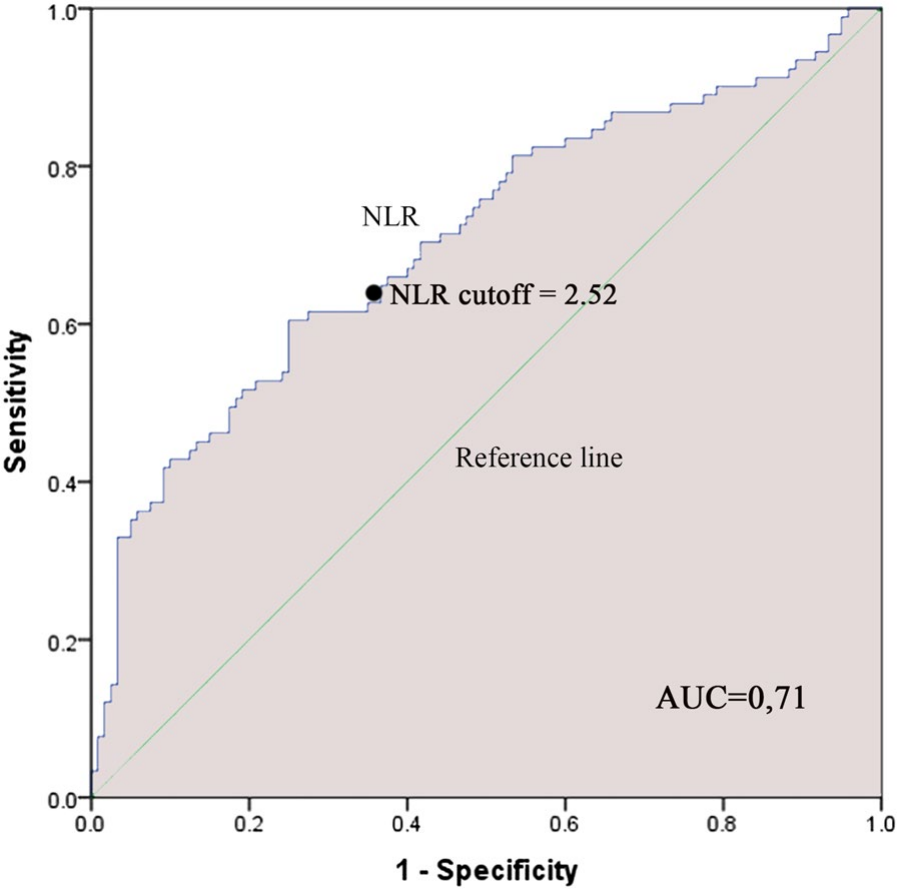
ROC Curve of NLR predictor cutoff of hospitalization of moderate to severe asthma pediatric patients. The ROC curve shows that the predictor cutoff of NLR for hospital admission is 2.52. Area under the curve was 0.71

There was significantly difference in hospitalized and non-hospitalized patients based on mean NLR ($4.96 \pm 4.27$ vs. $2.29 \pm 2.5$, P < 0.001). Using ROC curve analysis (Fig. 2) revealed that the indicator cutoff of NLR for hospitalization was 2.52 (AUC 0.71, sensitivity 63.7%, specificity 63.4%). According to this cutoff value, patients were categorized into a group of 102 patients with a high NLR and another of 109 patients with a low NLR.

In hospitalized patients with an NLR < 2.52, the age was significantly lower than patients with a higher NLR ($3.67 \pm 3.68$ vs. $6.02 \pm 3.36$, $P$ $< 0.001$). No significant correlation was found between eosinophil count and NLR ($\rho$ = 0.079, *P* = 0.454) in hospitalized patients, while there was a significant positive correlation between NLR and age ($\rho$ = 0.501, *P* < 0.001).

### Independent predictors of hospitalization

The univariate regression analysis showed female gender, infection as a cause for exacerbation, not using an asthma action plan, a blood eosinophil count $\ge$ 298 and an NLR $\ge$ 2.52 were associated with hospitalization. Multivariate analysis revealed that not using an asthma action plan (OR 2.22, 95% CI 1.09–4.49; *P* = 0.027), a blood eosinophil count $\ge$ 298 (OR 8.79, 95% CI 4.44–17.4; *P* < 0.001) and an NLR $\ge$ 2.52 (OR 2.13, 95% CI 1.09–4.14; *P* = 0.027) are significant factors predicting hospitalization in patients with asthma exacerbation (Table 3).

**Table 3:** Univariate and multivariate analyses to determine predictors of hospitalization in pediatric patients with asthma exacerbation

	Univariate analysis		Multivariate analysis	
	Crude OR (95% CI)	*P*	Adjusted OR (95% CI)	*P*
Gender, female	1.87 (1.07–3.28)	0.029	1.96 (0.98–3.91)	0.057
Not using an asthma action plan	2.15 (1.2–3.84)	0.01	2.22 (1.09–4.49)	0.027
Atopy status	1.13 (0.63–2.02)	0.674	–	–
Cause of the exacerbation				
Infection versus non-infectious cause	1.53 (0.85–2.73)	0.153	1.65 (0.81–3.36)	0.169
Allergen versus non-allergenic cause	0.58 (0.19–1.72)	0.323	–	–
Non-adherence to prescribed therapy versus adherence	0.87 (0.45–1.68)	0.671	–	–
Duration of asthma				
< 1 year versus > 1 year	0.69 (0.37–1.27)	0.233	–	–
1–2 years versus < 1 year and > 2 years	1.04 (0.57–1.87)	0.904	–	–
> 2 years versus < 2 years	0.68 (0.34–1.37)	0.28	–	–
Biomarkers				
Blood eosinophil count $\ge$ 298 versus < 298	8.87 (4.73–16.62)	< 0.001	8.79 (4.44–17.4)	< 0.001
NLR $\ge$ 2.52 versus < 2.52	3.04 (1.72–5.35)	< 0.001	2.13 (1.09–4.14)	0.027

## Discussion

Asthma, being the most common chronic disease amongst children [1], has a high healthcare cost for exacerbation and hospital admission [3]. Since asthma severity is known to have correlations with the level of blood eosinophils and sputum eosinophils, patients with eosinophilic asthma concern healthcare system particularly [7]. In spite of the attempts to recognize and lessen exposure to asthma triggers, develop treatment plans, and treatment of comorbidities, some patients suffer from refractory asthma. Efforts to characterize these patients have resulted in the concept of different phenotypes of severe asthma that has raised the hopes of improvement in the outcomes of each asthma patient by treatments fit for the particular phenotype [20–23]. For instance, anti-interleukin (IL)-5 specifically targets patients with a phenotype of Type-2 cytokine/eosinophil. Exacerbations in severe asthma patients, who have blood eosinophil counts of 150 cells/µL or more, are reduced with Mepolizumab, a monoclonal antibody to IL-5 [24]. Considering all this, blood eosinophil level, which is easier to acquire compared to sputum eosinophil count, is a good biomarker for assessing severe asthma as it enables us to define a phenotype, evaluate the patient’s response to the treatments, and observe the course of the disease. It is also cheap and reliable [25]. These prompted the performance of a study on blood biomarkers and their predictive role of hospitalization which could help the health policy committee with patient care management and prevention strategies for asthma exacerbation. Our study results revealed that, blood eosinophil count and NLR can be used as indicators for hospitalization in patients with moderate to severe asthma exacerbations.

In this study, a blood eosinophil count $\ge$ 298 (OR 8.79, 95% CI 4.44–17.4; *P* < 0.001) was associated with hospital admissions. Previous studies have investigated on blood eosinophil count in asthma patients using different methods. 3 studies conducted in United Kingdom (UK) and United States (US) strongly supported the increase in blood eosinophil counts $\ge 400$ cells/µL in asthma exacerbations [14, 15, 26]. Casciano et al. [16] and Makela et al. [27] have shown the association between elevated eosinophil counts and hospital admissions. In contrast to the current study, Pola-Bibian et al. [28] in a retrospective non-interventional cohort study carried out in Spain have demonstrated hospital admissions are significantly lower in patients who have a number of eosinophils of $\ge 400$ cells/µL in the blood after an ED visit. This discrepancy between results of the studies may be attributed to different ages in study population and different phenotypes of asthma. In our study, presence of eosinophilia was shown to be significantly higher among hospitalized patients than those reported in similar studies both in adults and children [15, 16, 29]. Higher rate of eosinophilia in this study can be due to different phenotypes of asthma involving different severity and prognosis. Phenotypes of asthma were not specified in our patients and there is no study conducted in Iran about phenotypes of asthma in different ages, although some studies revealed that eosinophilic phenotype is more common in late-onset asthma [23].

In our study, occurrence of more severe asthma exacerbations leading to hospitalization was found to be associated with higher NLR (OR 2.13, 95% CI 1.09–4.14; *P* = 0.027). NLR is a marker of inflammation and paediatric asthma exacerbations are mostly triggered by viral infections causing airway inflammation, and this explanation is in line with our finding. Machimaro, et al. [18] found increased likelihood of severe asthma exacerbations as a result of high NLR. To date, no study has evaluated NLR as a predictor for hospitalization in asthma exacerbation. Seemingly, further longitudinal studies are required for investigation on predictive factors in hospitalization due to asthma exacerbation.

Another finding of this study was the association of not using an asthma action plan with hospital admissions (OR 2.22, 95% CI 1.09–4.49; *P* = 0.027). Parents of children with asthma should be educated about the disease, the medications and proper way to use them, and what to do in an emergency. Previous studies [30, 31] showed that the written asthma action plan was helpful in educating the patients about their disease, increasing their quality of life, and boosting patients’ confidence in management of their asthma.

Results of the current study showed a positive correlation between age and NLR ($\rho$ = 0.503, $P < 0.001$). This means where age increases, NLR also increases. Considering lower neutrophil count and higher lymphocyte count in early ages [32], this correlation could be explained to some extent. Moreover, Li et al. [33] showed a positive correlation between NLR and age in a healthy population.

Nacaroglu et al. [34] in a study conducted in Turkey, studied 54 children admitted in hospital with asthma exacerbation, their results showed a value of $4.9 \pm 8.1$ for mean NLR, which is similar to our finding as in our study (mean NLR was equal to $4.96 \pm 4.26$ in hospitalized patients).

Huang et al. [35] and also De Jager et al. [36] showed NLR increased in patients with community-acquired pneumonia. In contrary to those studies, no significant difference was observed between NLR and concomitant pneumonia in our study. This could be due to low number of patients with concomitant pneumonia in our study and different study population, as in our study, only asthma patients were studied not those with pneumonia.

Our results indicated that, antibiotic therapy had a significantly higher percentage among hospitalized patients (60.4% vs. 19%, $P < 0.001$). Most used antibiotics were macrolides administered for 20 patients (36.7% of antibiotics). To the best of our knowledge, the most common cause of pediatric asthma exacerbation is viral infection which has no need for antibiotic therapy. Furthermore, in most of cases who were treated with antibiotic, antibiotic was discontinued within first 48 h due to general well-being and normal lab data. It seems the majority of received antibiotics was unnecessary, and overuse of antibiotics in asthma patients is a worldwide issue [37, 38] contributing to bacterial antibiotic resistance. Moreover, adverse reaction to antibiotics and increased health care costs caused by inappropriate use of antibiotic therapy should be considered. Further studies are needed for evaluation of causes and proper solutions for inappropriate antibiotic therapy.

There were some limitations in our study. Main limitation was that, there was not any significant data about phenotypes of asthma in Iran and they were not checked in our patients before or after the exacerbations. Study on presence of eosinophil and NLR in bronchial system is more accurate and reliable compared to their presence in the blood, which can be considered for further studies especially regarding phenotyping of asthma.

## Conclusions

Blood eosinophil count and NLR were found to be higher in the patients hospitalized due to asthma exacerbation. These markers can be predictors for hospitalization in asthma exacerbations in addition to clinical judgment.

## Supplementary Information

**Additional file 1.** Patients' data on demographic characteristics and laboratory findings. 

## Data Availability

The dataset supporting the conclusions of this article is included within the article and its additional file.
